# TRPV1 Antagonist DWP05195 Induces ER Stress-Dependent Apoptosis through the ROS-p38-CHOP Pathway in Human Ovarian Cancer Cells

**DOI:** 10.3390/cancers12061702

**Published:** 2020-06-26

**Authors:** Yi-Yue Wang, Kyung-Tae Lee, Myong Cheol Lim, Jung-Hye Choi

**Affiliations:** 1 Department of Life and Nanopharmaceutical Sciences, Kyung Hee University, Seoul 02447, Korea; yiyojanuary@naver.com (Y.-Y.W.); ktlee@khu.ac.kr (K.-T.L.); 2 College of Pharmacy, Kyung Hee University, Seoul 02447, Korea; 3 Division of Tumor Immunology, Center for Gynecologic Cancer & Center for Clinical Trials, National Cancer Center, Goyang 10408, Korea; gynlim@gmail.com

**Keywords:** DWP05195, TRPV1 antagonist, ovarian cancer, ER stress, CHOP, ROS

## Abstract

In addition to their analgesic activity, transient receptor potential vanilloid 1 (TRPV1) agonists and antagonists demonstrate profound anti-cancer activities in various human cancers. In the present study, we investigated the anti-cancer activity of a novel TRPV1 antagonist, DWP05195, and evaluated its molecular mechanism in human ovarian cancer cells. DWP05195 demonstrated potent growth inhibitory effects in all five ovarian cancer cell lines examined. DWP05195 induced apoptosis through the activation of caspase-3, -8, and -9. DWP05195 induced C/EBP homologous protein (CHOP) expression and endoplasmic reticulum (ER) stress. Sodium phenylbutyrate (4-PBA), an ER-stress inhibitor, and CHOP knockdown significantly suppressed DWP5195-induced cell death. DWP05195-enhanced CHOP expression stimulated intrinsic and extrinsic apoptotic pathways through the regulation of Bcl2-like11 (BIM), death receptor 4 (DR4), and DR5. DWP05195-induced cell death was associated with increased reactive oxygen species (ROS) levels and p38 pathway activation. Pre-treatment with the antioxidant N-acetyl-L-cysteine (NAC) significantly suppressed DWP05195-induced CHOP expression and p38 activation. Inhibition of NADPH oxidase (NOX) through p47phox knockdown abolished DWP05195-induced CHOP expression and cell death. Taken together, the findings indicate that DWP05195 induces ER stress-induced apoptosis via the ROS-p38-CHOP pathway in human ovarian cancer cells.

## 1. Introduction

Ovarian cancer is one of the most lethal gynecological malignancies, with around 300,000 women diagnosed and more than 184,000 related deaths in 2018 worldwide [1]. Due to ambiguous symptoms during the early stage and a lack of effective biomarkers, most women are diagnosed at an advanced stage [2,3]. The overall 5-year survival rate for advanced ovarian cancer is approximately 30% [4]. Cytoreductive surgery and platinum- and taxane-based chemotherapy are still the standard therapy in women with ovarian cancer [5]. Both platinum and taxane commonly cause several side effects and complications. Furthermore, chemoresistance is a major cause of recurrence and death. Thus, a novel therapeutic agent to treat ovarian cancer is urgently needed.

The transient receptor potential vanilloid 1 (TRPV1) is a nonselective ligand-gated cation channel with high calcium permeability and can be activated by various exogenous and endogenous stimuli, such as low pH, noxious heat, and vanilloids [6]. Moreover, it is well known that TRPV1 plays a role in pain modulation and can be targeted for pain management [7]. Interestingly, recent studies have revealed that TRPV1 antagonists, as well as TRPV1 agonists, possess anti-cancer activities in various types of human cancer cells, in addition to their analgesic activity. For example, TRPV1 antagonists such as capsazepine induce apoptosis and suppress tumor growth in colorectal and oral cancer [8,9,10]. However, the molecular mechanism of action of the TRPV1 antagonists, underlying the anti-cancer activities, remains poorly characterized. Additionally, according to our knowledge, the effect of these drugs has not been reported in human ovarian cancer.

DWP05195, a novel TRPV1 antagonist, inhibits the pain signal transduction triggered by capsaicin and other typical TRPV1 agonists [11]. Reportedly, this drug shows an analgesic effect in both nerve injury and diabetic neuropathy animal models. However, its effect on other diseases, including cancer, remains unexamined. Thus, we investigated the anti-cancer activity of DWP05195 in this study, as well as the potential underlying mechanism of action in human ovarian cancer cells.

## 2. Results

### 2.1. DWP05195 Stimulates Caspase-Dependent Apoptosis in Human Ovarian Cancer Cells

**Figure 1:**
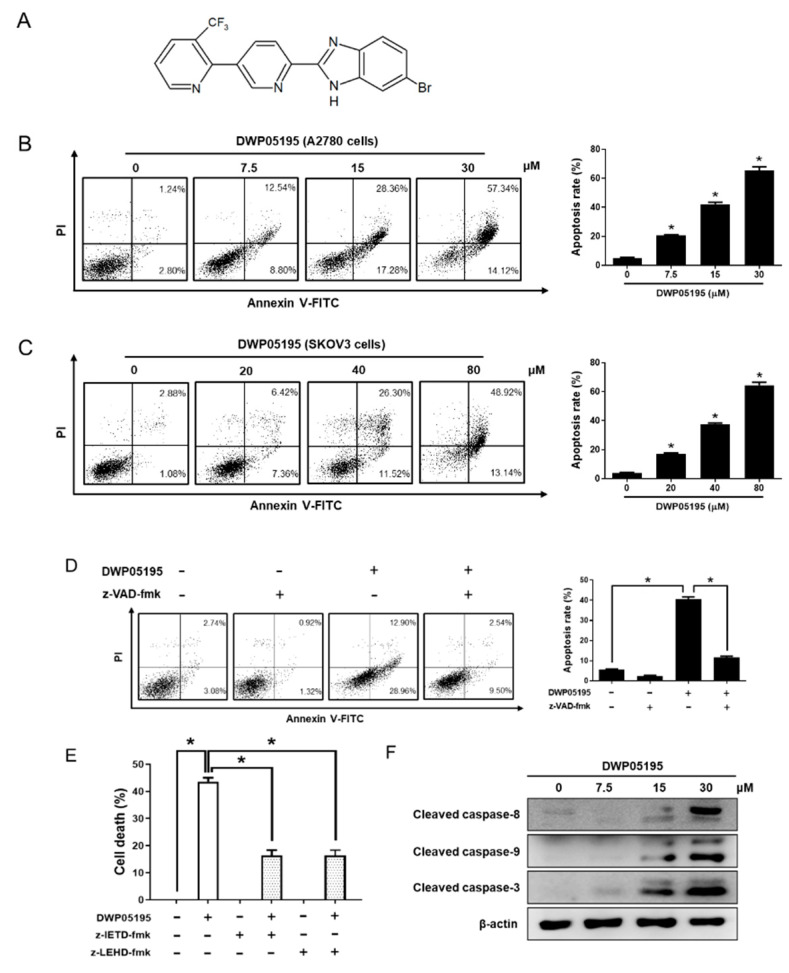
Effect of DWP05195 on apoptosis in A2780 and SKOV3 cells. (**A**) Chemical structure of DWP05195. (**B**) A2780 cells were treated with 0, 7.5, 15, and 30 µM of DWP05195 for 48 h. (**C**) SKOV3 cells were treated with 0, 20, 40, and 80 µM of DWP05195 for 48 h. On the day of harvest, A2780 or SKOV3 cells were co-stained with propidium iodide (PI) and Annexin V-FITC. * *p* < 0.05 compared with control. (**D**) A2780 cells were pre-treated with broad caspase inhibitor, z-VAD-fmk (50 µM), and then treated with DWP05195 (15 µM). PI/Annexin V-FITC staining assay was performed to determine apoptosis. (**E**) A2780 cells were pre-treated with caspase-8 inhibitor, z-IETD-fmk (50 µM) and caspase-9 inhibitor, z-LEHD-fmk (75 µM), and then treated with DWP05195 (15 µM). MTT assay was performed to determine cell viability. (**F**) A2780 cells were treated with 0, 7.5, 15, and 30 µM of DWP05195 for 48 h. Cleaved caspase-8, caspase-9, and caspase-3 levels were determined by Western blotting. Results are representative of at least three independent experiments. * *p* < 0.05. Uncropped blots of Figure 1F are shown in Figure S1.

DWP05195 (Figure 1A) demonstrated a growth inhibitory effect on the five human ovarian cancer cell lines (A2780, SKOV3, OVCAR3, TOV-21G, and Hey8A) used in this study (Table 1).

The Annexin V-FITC staining assay was performed to evaluate whether the growth-inhibitory effect of DWP05195 is associated with the induction of apoptosis. In A2780 and SKOV3 cells, DWP05195 dose-dependently enhanced the population of Annexin V-positive cells in the right quadrants of the flow cytometry graphs (Figure 1B,C). Additionally, we determined whether caspases were involved in DWP05195-induced apoptosis. DWP05195-induced apoptosis was significantly inhibited in the presence of a broad caspase inhibitor, z-VAD-fmk (Figure 1D), suggesting that caspases mediate DWP05195-induced apoptosis in human ovarian cancer cells. Additionally, caspase-8 inhibitor z-IETD-fmk and caspase-9 inhibitor z-LEHD-fmk markedly reversed the inhibitory effect of DWP05195 on cell viability (Figure 1E). Moreover, Western blot analysis revealed that DWP05195 activated caspases-8 and -9 (Figure 1F). These findings suggest that, in human ovarian cancer cells, DWP05195 induces apoptosis via both intrinsic and extrinsic apoptotic pathways.

### 2.2. ER Stress is Involved in DWP05195-Induced Apoptosis

**Figure 2:**
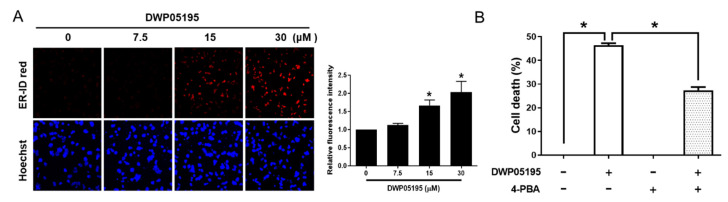
Involvement of endoplasmic reticulum (ER) stress in DWP05195-induced apoptosis. (**A**) A2780 cells were treated with 0, 7.5, 15, and 30 µM of DWP05195 for 24 h, and then analyzed using a confocal fluorescence microscope. Fluorescence intensity was analyzed with the ImageJ software. (**B**) Following pre-treatment with ER stress inhibitor, 4-PBA (500 µM), A2780 cells were treated with DWP05195 (15 µM). MTT assay was performed to determine cell viability. Results are representative of at least three independent experiments. * *p* < 0.05.

Capsazepine, a prototype TRPV1 antagonist, reportedly possesses anti-cancer activity and induces endoplasmic reticulum (ER) stress in several cancer cells [8,10]. Thus, we examined whether the novel TRPV1 antagonist, DWP05195, also induces ER stress in human ovarian cancer cells. Flow cytometric and fluorescence microscopic analysis demonstrated that DWP05195 increases ER-specific fluorescence intensity, indicating ER expansion, a hallmark of activated ER stress (Figure 2A). To further confirm the involvement of ER stress in DWP05195-induced apoptosis, we examined whether an ER stress inhibitor, 4-PBA (4-phenylbutyric acid) affected cell death (Figure 2B). In human ovarian cancer cells, 4-PBA significantly alleviated DWP05195-induced cell death.

**Figure 3:**
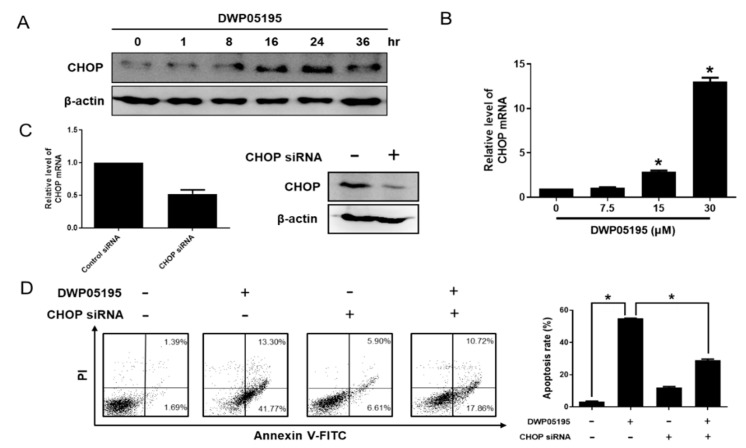
Involvement of C/EBP homologous protein (CHOP) in DWP05195-induced apoptosis. (**A**) A2780 cells were treated with DWP05195 (15 µM) for the indicated time and CHOP protein levels were detected using Western blotting. Uncropped blots of Figure 3A are shown in Figure S1 (**B**) A2780 cells were treated with DWP05195 for 24 h at the indicated concentrations and CHOP mRNA levels were determined by real-time RT-PCR. * *p* < 0.05 compared with control. (**C**) CHOP mRNA and protein levels after transfection with CHOP siRNA in A2780 cells were measured by real-time RT-PCR and Western blotting, respectively. Uncropped blots of Figure 3C are shown in Figure S1 (**D**) After the CHOP knockdown, cells were treated with 15 µM DWP05195 and used for analysis of apoptosis. Results are representative of at least three independent experiments. * *p* < 0.05.

C/EBP homologous protein (CHOP), a marker of ER stress, plays an important role in ER stress-induced apoptosis [12]. We observed that DWP05195 increased CHOP protein and mRNA levels in human ovarian cancer cells (Figure 3A,B). We further evaluated the role of CHOP in DWP05195-induced apoptosis. CHOP siRNA markedly suppressed CHOP mRNA and protein levels in A2780 cells (Figure 3C), and CHOP knockdown using the CHOP siRNA notably decreased the population of Annexin V-positive cells enhanced by DWP05195 treatment (Figure 3D). Collectively, these results indicate that enhanced CHOP expression and ER stress are required for DWP05195-induced apoptosis in human ovarian cancer cells.

### 2.3. CHOP Upregulation by DWP05195 Stimulates Both Intrinsic and Extrinsic Apoptosis Pathways

**Figure 4:**
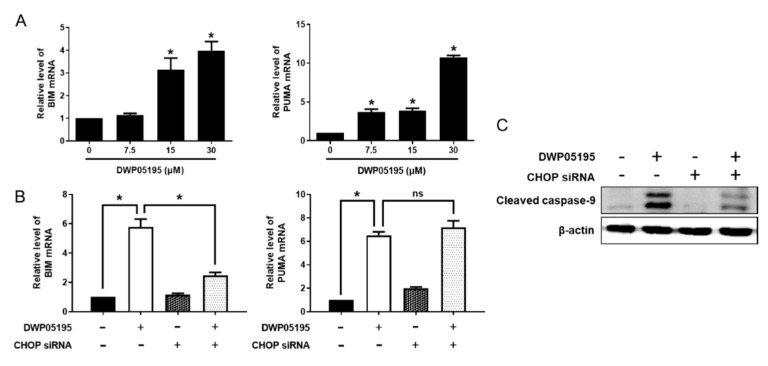
Involvement of CHOP in DWP05195-stimulated intrinsic apoptosis pathway. (**A**) A2780 cells were treated with 0, 7.5, 15, and 30 µM DWP05195 for 24 h. BIM (Bcl2-like11) and PUMA (p53 upregulated modulator of apoptosis) levels were determined by real-time RT-PCR. * *p* < 0.05 compared with control. (**B**) After the CHOP knockdown, BIM and PUMA levels were detected by real-time RT-PCR and (**C**) cleaved caspase-9 levels were detected by Western blotting Uncropped blots of Figure 4C are shown in Figure S1. Results are representative of at least three independent experiments. * *p* < 0.05.

CHOP can mediate apoptosis through intrinsic and extrinsic apoptotic pathways [13]. As DWP05195 induces the activation of both intrinsic and extrinsic initiator caspases, we first examined the effect of CHOP on the intrinsic pathway, also known as the mitochondrial pathway. As shown in Figure 4A, DWP05195 significantly increased the mRNA levels of BIM (Bcl2-like11) and PUMA (p53 upregulated modulator of apoptosis) in human ovarian cancer cells. CHOP knockdown effectively reduced the expression of BIM, but not that of PUMA (Figure 4B). Importantly, DWP05195-induced activation of the intrinsic initiator caspase, caspase-9, was markedly reversed by CHOP knockdown (Figure 4C).

**Figure 5:**
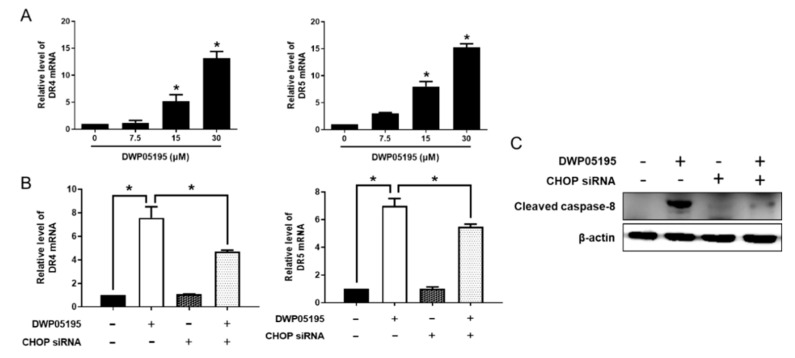
Involvement of CHOP in DWP05195-stimulated extrinsic apoptosis pathway. (**A**) A2780 cells were treated with 0, 7.5, 15, and 30 µM DWP05195 for 24 h. Death receptor 4 (DR4) and death receptor 5 (DR5) levels were determined by real-time RT-PCR. * *p* < 0.05 compared with control. (**B**) After the CHOP knockdown, DR4 and DR5 levels were detected by real-time RT-PCR and (**C**) cleaved caspase-8 levels were detected by Western blotting. Uncropped blots of Figure 5C are shown in Figure S1 Results are representative of at least three independent experiments. * *p* < 0.05.

These data suggested that CHOP expression, enhanced by DWP05195, stimulated the intrinsic apoptosis pathway through the upregulation of BIM, but not of PUMA. Furthermore, we confirmed the role of CHOP in DWP05195-induced apoptosis through the extrinsic death receptor pathway. As shown in Figure 5A, DWP05195 treatment increased the mRNA levels of death receptor 4 (DR4) and DR5 in human ovarian cancer cells. CHOP knockdown effectively reduced the expression of DR4 and DR5, enhanced by DWP05195 treatment (Figure 5B). Furthermore, the DWP05195-induced activation of caspase-8, an extrinsic initiator caspase, was markedly reversed following CHOP knockdown (Figure 5C). These results confirmed that DWP05195 induces apoptosis by the upregulation of CHOP, which regulates both intrinsic and extrinsic apoptosis pathways via BIM and DR4/5.

### 2.4. DWP05195 Induces ER Stress-Dependent Apoptosis by ROS Upregulation and p38 Activation

DWP05195 is a novel TRPV1 antagonist possessing inhibitory effects on pain signal transduction activated by TRPV1 agonists such as capsaicin [11]. The TRPV1 channel is highly permeable to calcium ions, which play a critical role in several cellular processes, including apoptosis and proliferation of cancer cells [14]. Accordingly, we examined whether calcium signaling through the TRPV1 channel is involved in DWP05195-induced apoptosis in human ovarian cancer cells. First, intracellular calcium levels were measured after DWP05195 treatment (Figure S2). As expected, capsaicin, the TRPV1 agonist, induced calcium influx in ovarian cancer cells, and DWP05195 reversed the influx. However, treatment with DWP05195 (15 μM) alone, which can induce apoptosis in ovarian cancer cells, did not cause a prompt change of the levels of intracellular calcium, suggesting that a direct modulation of calcium-conducting channels may not be involved in DWP05195-induced apoptosis.

**Figure 6:**
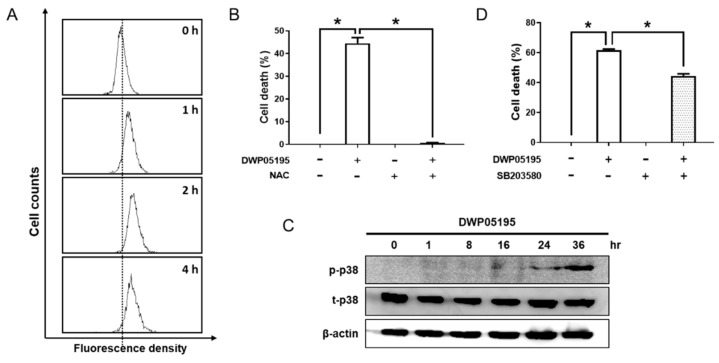
Involvement of ROS and p38 in DWP05195-induced apoptosis. (**A**) A2780 cells were treated with DWP05195 (15 µM) for the indicated time. The cells were stained with dihydro-fluorescein diacetate (DCFH-DA) and analyzed by flow cytometry. (**B**) A2780 cells were pre-treated with N-acetyl-L-cysteine (NAC) (7.5 mM), and then treated with DWP05195 (15 µM). MTT assay was performed to determine cell viability. (**C**) A2780 cells were treated with DWP05195 (15 µM) for the indicated time. Phosphor-p38 and total p38 levels were determined by Western blotting. Uncropped blots of Figure 6C are shown in Figure S1 (**D**) A2780 cells were pre-treated with p38 inhibitor SB203580 (0.5 µM) and then treated with DWP05195 (15 µM). MTT assay was performed to determine cell viability. Results are representative of at least three independent experiments. * *p* < 0.05.

Considering that intracellular ROS and mitogen-activated protein kinase (MAPK) signaling are implicated in cancer cell apoptosis [15], we investigated the effect of DWP05195 on the intracellular levels of ROS. In human ovarian cancer cells, DWP05195 markedly induced ROS production (Figure 6A), and DWP05195-induced cell death was inhibited in the presence of the antioxidant N-acetyl-L-cysteine (NAC) (Figure 6B). These data suggested that enhanced intracellular ROS levels are required for DWP05195-induced apoptosis in human ovarian cancer cells. Additionally, we observed that DWP05195 increased the activation of p38 MAPK (Figure 6C) and the p38 inhibitor, SB203580, partially reduced DWP05195-induced cell death (Figure 6D).

**Figure 7:**
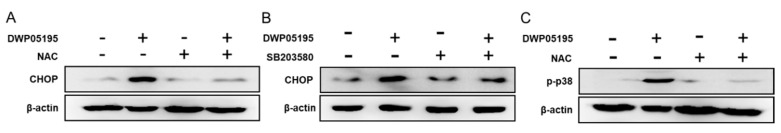
The involvement of ROS levels and p38 activation in CHOP expression. (**A**) A2780 cells were pre-treated with antioxidant NAC (7.5 mM) or (**B**) SB203580 (20 µM), and then treated with DWP05195 (15 µM) for 24 h. CHOP levels were determined by Western blotting. (**C**) A2780 cells were pre-treated with NAC (7.5 mM), and then treated with DWP05195 (15 µM) for 24 h. Phosphor-p38 levels were determined by Western blotting. Results are representative of at least three independent experiments. * *p* < 0.05. Uncropped blots of Figure 7 are shown in Figure S1.

Conversely, unlike p38, extracellular signal-regulated kinase (ERK1/2) and c-Jun N-terminal kinase (JNK) were not associated with DWP05195-induced cell death (Figure S3). We further investigated whether ROS acts upstream of p38 MAPK activation and ER stress induced by DWP05195. Both NAC and SB203580 markedly suppressed the expression of CHOP (Figure 7A,B). Additionally, NAC substantially inhibited the activation of p38 (Figure 7C). Collectively, the DWP05195-increased ROS levels mediated the ER-stress-dependent apoptosis, at least in part, through the p38 pathway.

### 2.5. DWP05195 Regulates the Intracellular ROS Levels Through NADPH Oxidase

**Figure 8:**
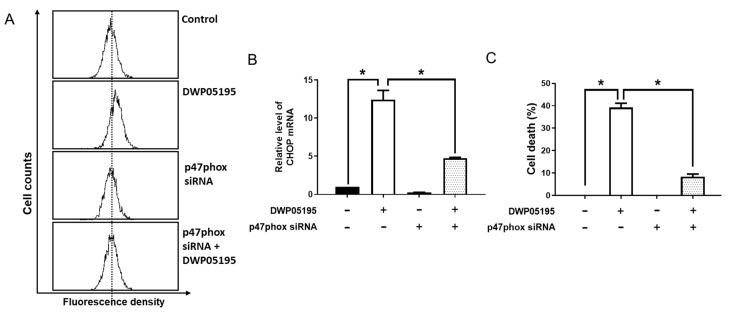
Involvement of NADPH oxidase activation in DWP05195-induced apoptosis. (**A**) A2780 cells were transfected with p47phox siRNA for 24 h and treated with DWP05195 (15 µM) for 2 h. The cells were stained with DCFH-DA and analyzed by flow cytometry. (**B**) After knockdown of p47phox, cells were treated with DWP05195 (15 µM) for 24 h. CHOP levels were measured by real-time RT-PCR. (**C**) After knockdown of p47phox, cells were treated with DWP05195 (15 µM). MTT assay was performed to determine cell viability. Results are representative of at least three independent experiments. * *p* < 0.05.

The transmembrane enzymes, NADPH oxidases (NOXs), are considered the main source of intracellular ROS in various cell types [16,17]. Interestingly, the functional interaction between NOXs and TRPVs has been implicated in various pathophysiological conditions [18,19,20]. Thus, we examined whether NOX is associated with DWP05195-induced ER stress and apoptosis in human ovarian cancer cells. Inhibition of NOX through the knockdown of NOX-regulatory-subunit, p47phox markedly suppressed DWP05195-induced ROS upregulation and CHOP expression (Figure 8A,B). Notably, p47phox knockdown significantly reversed DWP05195-induced cell death (Figure 8C). These data indicate that NOX is involved in DWP05195-induced ER stress and apoptosis via ROS regulation in human ovarian cancer cells.

## 3. Discussion

Reportedly, TRPV1 is expressed in various human cancer tissues [21,22,23,24,25,26], with TRPV1 expression found to be associated with lower survival in invasive breast carcinoma [27]. In melanoma tissues, decreased TRPV1 expression has been observed when compared to normal melanocytes, and TRPV1 overexpression inhibits melanoma growth via Ca^2+^ signaling. Collectively, these results suggest a potential role for TRPV1 in human cancers. We observed that TRPV1 is expressed in immortalized ovarian epithelial cells (IOSE80PC) and human ovarian cancer cells (Figure S3). SKOV3, A2780, OVCAR3, and HEY8A cells demonstrated higher TRPV1 expression than IOSE80PC cells, but not TOV-21G cells. Moreover, we analyzed data from TCGA (The Cancer Genome Atlas) and GEO (Gene Expression Omnibus; GSE14407) for ovarian cancer to assess whether TRPV1 expression is associated with prognosis and survival of ovarian cancer patients. However, no significant correlation was observed. These data indicate that human ovarian cancer cells express TRPV1; however, its expression may not be a potential diagnostic and prognostic marker for ovarian cancer.

Although some reports have shown the pro-tumor activity of capsaicin [28], accumulating evidence indicates that TRPV1 agonists have anti-cancer activities [29,30,31,32,33,34]. Capsaicin, the best-known TRPV1 agonist, stimulates apoptosis in pancreatic, urothelial, and renal cancer cells, and gliomas. In the case of ovarian cancer, one study has demonstrated the apoptosis-inducing effect of capsaicin using fatty-acid-conjugated lipid nanoparticles [35]. Furthermore, capsaicin reportedly inhibits the metastasis of thyroid cancer [32]. Resiniferatoxin (RTX), another potent TRPV1 agonist, induces apoptosis and decreases cancer cell growth in pancreatic cancer cells [36]. Primarily, TRPV1 agonist-induced anti-cancer activities seem to involve TRPV1 and its calcium signaling. For example, capsaicin induces caspase activation and apoptosis through increased Ca^2+^ influx in glioma cells; these events were markedly inhibited by TRPV1 antagonist [34]. In melanoma cells, TRPV1 induces Ca^2+^ influx to regulate p53 activation via the calcineurin-ATF3 transcriptional cascade [37]. Interestingly, TRPV1 antagonists such as capsazepine also induced cell death in several cancers [8,9,10]. Notably, it remains controversial whether the TRPV1 antagonist-induced anti-cancer activities are associated with TRPV1 and its calcium signaling. Teng et al. and Huang et al. have reported that capsazepine increases intracellular calcium levels and cytotoxicity in human osteosarcoma and prostate cancer cells, respectively [38,39]. In contrast, Sung et al. have suggested that capsazepine potentiated TRAIL-induced apoptosis, in a TRPV1 independent manner, by TRAIL upregulation in colorectal cancer cells [10]. Furthermore, capsazepine reportedly induces TRPV1-independent apoptosis in oral squamous cell carcinoma (OSCC) [40]. In the present study, we found that the TRPV1 agonist capsaicin has little cytotoxic activity in most cancer cells including ovarian cancer cells (Table S1). In contrast, DWP05195 revealed a cytotoxic activity in other types of cancer cells including neuroblastoma and colon and endometrial cancer (Table 1 and Table S2), suggesting that DWP05195 may induce apoptotic cell death not only in ovarian cancer cells but also in other types of cancer cells. However, the cytotoxic activity of DWP05195 was not strongly associated with the levels of TRPV1 in the cancer cells (Figures S4 and S5). Moreover, to determine whether the DWP05195-induced apoptosis is mediated through the TRPV1 receptor, we examined cell death after treatment with DWP05195 and the TRPV1 agonist, capsaicin, alone or in combination. Capsaicin alone markedly increased intracellular calcium levels and induced cell death (Figures S2 and S6), which is consistent with previous findings. However, capsaicin failed to reverse the effect of DWP05195 on cell death (Figure S6). These data indicate that, in human ovarian cancer cells, DWP05195-induced cell death is not mediated through TRPV1-induced calcium signaling. Considering that TRPV1 is redox-sensitive, there is a possibility that ROS induced by DWP05195 may counteract the antagonist effect of DWP05195 in the ovarian cancer cells.

A wide variety of physiological and biochemical stimuli, such as oxidative stress, induce ER stress [41,42]. If cells fail to overcome ER stress and restore homeostasis to survive, this will result in apoptosis. Upon ER stress, the expression of the transcription factor CHOP is mainly upregulated by ER-stress-sensor proteins, which play a crucial role in ER-stress-induced apoptosis [43,44]. CHOP regulates the BH3-only proteins (such as BID, BIM, and PUMA), triggering the intrinsic apoptotic pathway by regulating the Bcl-2 family proteins, which play a pivotal role in the mitochondrial membrane potential (MMP) (Δψm) [45,46,47]. Conversely, CHOP mediates the extrinsic apoptotic pathway by directly regulating the expression of death receptors and activating the caspase-8 cascade [12]. CHOP also has been suggested to induce apoptosis via the activation of other downstream regulatory pathways [13]. Here, we demonstrated that DWP05195 induced ER-dependent apoptosis by CHOP upregulation through both intrinsic and extrinsic pathways in human ovarian cancer cells. This finding is consistent with previous reports demonstrating that some TRPV1 antagonists induce cancer cell death by inducing ER stress [8,10].

Notably, ROS production is involved in multiple events in numerous diseases, including cancer [48,49]. Several anti-cancer agents stimulate apoptosis via ROS production in human ovarian cancer cells [50,51]. In response to physiological and pharmacological stimuli, ROS can be produced by oxidases such as NADPH oxidases (NOXs), which are considered the main source of ROS in cells [16,17]. NOXs are expressed in human cancer cell lines with functions in cell proliferation and tumorigenesis [52]. Reportedly, several studies have demonstrated that NOX activation promotes apoptosis in cancer cells [53]. For instance, NOX is upregulated by TGFβ, which is associated with its pro-apoptotic activity in liver cancer cells [54]. In this study, we demonstrated the involvement of NOX in DWP05195-induced ROS upregulation, which resulted in the apoptosis of ovarian cancer cells. Notably, the effects of vanilloids, including TRPV1 agonist and antagonist, on NOX oxidase and the interaction between NOXs and TRPV1 have been previously demonstrated [18,19,20,55]. These findings suggest that DWP05195 affects the activity of NOX oxidase, directly or through TRPV1. The detailed molecular mechanism of action underlying the ROS regulation mediated by DWP05195 needs to be elucidated. Additionally, considering the partial effect of NOX inhibition on DWP05195-induced apoptosis, further investigations are crucial to explore other potential mechanisms by which DWP05195 induces ROS.

Notably, a variety of signaling pathways can be activated by the increase in ROS, including the MAPK pathway [56]. The MAPK pathway, including ERK1/2, JNK, and p38 MAPK, is ordinarily activated and functions as a regulator in the development and progression of cancer [57,58,59,60]. Generally, JNK and p38 MAPK are stress-activated MAPK pathways, mediating downstream stress responses that lead to cell death, while ERK is activated by growth factors and is related to cell proliferation [61,62,63]. We have demonstrated that DWP05195-induced ROS resulted in p38 activation, which was partially associated with ER stress and apoptosis in human ovarian cancer cells.

Our findings suggest that DWP05195 can be consider a potential therapeutic for ovarian cancer. However, the relatively high dose of DWP05195 used in this study would exceed therapeutic clinical use. The in vivo effect of DWP05195 on ovarian cancer should be further investigated.

## 4. Materials and Methods 

### 4.1. Cell Lines and Materials

Human ovarian cancer cell lines (A2780, SKOV3, OVCAR3, TOV-21G, and Hey8A) were originally obtained from American Type Culture Collection (ATCC; Manassas, VA, USA). RPMI 1640 medium, FBS, streptomycin sulphate, and penicillin were obtained from Life Technologies Inc. (Grand Island, NY, USA). DWP05195 used for this study was kindly supplied by Daewoong Pharmaceutical Co., Ltd. (Seoul, South Korea). N-acetyl-L-cysteine (NAC) was purchased from Sigma Chemical (St. Louis, MO, USA). All inhibitors for caspases were purchased from Calbiochem (Bad Soden, Germany).

### 4.2. MTT Assay

Cells were cultured in Roswell Park Memorial Institute (RPMI) 1640 medium containing 5% fetal bovine serum (FBS), streptomycin sulphate (100 μg/mL), penicillin (100 U/mL) at 37 °C in 5%. To analysis cell viability, 3-(4,5-dimethylthiazol-2-yl)-2,5-diphenyl-tetrazolium bromide (MTT) assay was carried out. Cells were plated in a 96-well plate (5 × 10^4^ cells/well) and incubated overnight to allow adhesion of the cells. After treatment with various concentrations of DWP05195, the cells were incubated for 48 h. Then, 50 μL of MTT solution (1 mg/mL stock solution) was added into each well and the plate was incubated for another 3 h. Formazan crystal formed in the cells was dissolved in DMSO after removal of culture medium. The absorbance at 540 nm was measured in a microplate reader (SpectraMax; Molecular Devices, Sunnyvale, CA, USA).

### 4.3. Annexin V and Propidium Iodide (PI) Double Staining for Apoptosis Analysis

Apoptosis was examined using Annexin V and PI double staining using ApoScan kit (Annexin V-FITC Apoptosis Detection kit) (Biobud Inc., Gyunggido, South Korea). Briefly, the cells were harvested and rinsed twice with cold PBS. Then, the cells were suspended in the binding buffer (10 mM HEPES/NaOH, 2.5 mM CaCl_2_, 140 mM NaCl, pH 7.4). Next, the cells were stained with PI for 5 min and FITC-conjugated Annexin V for 15 min in a dark place. Guava^®^ easyCyte flow cytometry (EMD Milipore, Bilerica, MA, USA) was used to analyze the mixture.

### 4.4. Western Blot Analysis

Cells were rinsed twice with cold PBS and resuspended with a protein lysis buffer (Intron Biotechnology, Seoul, South Korea). After protein quantification using Bradford assay, the lysate was denatured with the SDS-PAGE sample buffer followed by 5 min boiling at 95 °C. Total protein (30 μg) was used for SDS-PAGE and the separated proteins were blotted onto polyvinylidene difluoride (PVDF) membrane from the gel. The membrane was post-coated with 5% skimmed milk in Tris-buffered saline (Boster Biological Technology Ltd., Wuhan, China) containing Tween-20 for 1 h. After incubation overnight at 4 °C with the diluted corresponding primary antibodies, the membrane was incubated with optimal dilution of the appropriate horseradish peroxidase-linked secondary antibody for 2 h at room temperature. Anti-caspase-8 was purchased from BD Biosciences (San Jose, CA, USA). Caspase-3 and β-actin antibodies were obtained from Santa Cruz Biotechnology (Santa Cruz, CA, USA). Antibodies for caspase-9, phospho-p38 MAPK, and CHOP were from Cell Signaling (Beverly, MA, USA). Secondary antibodies were obtained from The Jackson Laboratory (West Grove, PA, USA). Enhanced chemiluminescence (ECL) kit (EMD Millipore, Billerica, MA, USA) was used for visualization of immunoreactive bands.

### 4.5. Determination of Endoplasmic Reticulum Stress

Endoplasmic reticulum (ER)-specific fluorescence intensity, indicating ER expansion, a hallmark of activated ER stress, was measured using the ER-ID Red assay kit (Enzo Life Science, Farmingdale, NY, USA). Cells were seeded in a 60 mm or 96-well plate culture dish. After a 24 h incubation, DWP05195 was diluted with the culture medium and added to the cells. Following another 24 h incubation, 100 µL 1X Assay Buffer with 1 µL of ER-ID Red Detection Reagent and 1 µL of Hoechst 33342 Nuclear Stain was added into each wells or dishes, and the cells were incubated for 30 min at 37 °C. The fluorescent intensity was analyzed by flow cytometry and cells were imaged using a confocal fluorescence microscope.

### 4.6. Detction of Intracellular Reactive Oxygen Species

The intracellular levels of reactive oxygen species (ROS) were determined using the fluorescent probe dihydro-fluorescein diacetate (DCFH-DA) (Santa Cruz Biotechnology, Santa Cruz, CA, USA). After treatment with DWP05195 at the desired time intervals, the cells were collected by centrifugation, suspended in PBS, and loaded with 20 μM DCFH-DA at 37 °C for 30 min. The fluorescent intensity of the formed DCF was analyzed by flow cytometry.

### 4.7. RNA Inteference for Gene Knockdown

For small interfering RNA (siRNA)-mediated knockdown of genes (TRPV1, CHOP, and p47phox), the cells were transfected with either the targeting or control siRNA using lipofectamine (Invitrogen; Carlsbad, CA, USA). The siRNAs were purchased from Bioneer Technology (Daejeon, South Korea). Briefly, the cells were seeded in 6-well plates and grown for 24 h before transfection. Each siRNA was mixed with lipofectamine in serum-free Opti-MEM (Life Technologies Inc., Grand Island, NY, USA) at a final concentration of 50 nM. The transfection mixtures were incubated for 10 min at room temperature before adding to the cells. After 24 h incubation, the transfected cells were used for the experiment.

### 4.8. Real-Time Reversed Transcription-PCR

In accordance with the manufacturer’s protocol, total RNA (500 ng) was reverse transcribed into complementary DNA (cDNA) using first-strand cDNA synthesis kit (Amersham Pharmacia Biotech, Oakville, Canada) following RNA extraction using Easy Blue^®^ kits (Intron Biotechnology, Seoul, South Korea). The primers used for this study were as follows: for CHOP sense primer, 5′-TTG CCT TTC TCC TTC GGG AC-3′ and antisense primer, 5′-CAG TCA GCC AAG CCA GAG AA-3′; for BIM sense primer, 5′-CTT CCA TGA GGC AGG CTG AA-3′ and antisense primer, 5′-ACC ATT CGT GGG TGG TCT TC-3′; for PUMA sense primer, 5′-TGA AAT TTG GCA TGG GGT CGT C-3′ and anti-sense primer, 5′-CTC CCT GGG GCC ACA AAT CT-3′; for DR4 sense primer, 5′-AAG TGC ATG GAC AGG GTG TG-3′ and anti-sense primer, 5′-GAG TCT GCG TTG CTC AGA ATC-3′; for DR5 sense primer, 5′-GTC CCA GAG GGA TGG TCA AG-3′ and anti-sense primer, 5′-CCC ACT GTG CTT TGT ACC TGA-3’; for p47phox sense primer, 5′-TTG AGA AGC GCT TCG TAC CC-3′ and anti-sense primer, 5′-CGT CAA ACC ACT TGG GAG CT-3′; and for TRPV1 sense primer, 5′-GTA CAC ACC TGA TGG CAA GG-3′ and anti-sense primer, 5′-TCT TCG TTG ATG ATG CCC AC-3′. Reverse-transcription (RT)-PCR was carried out using the SYBR Premix Ex Taq™ Kit and Thermal Cycler Dice Real-Time PCR System (TaKaRa, Kyoto, Japan). For each gene, the average cycle threshold (Ct) value of each triplicate measurements was converted to relative quantity data. Gene expression was normalized with respect to β-actin.

### 4.9. Statistical Analysis

Data are presented as mean ± SD. All statistical parameters were calculated using GraphPad Prism 5.0 (GraphPad Software, Inc., La Jolla, CA, USA). Results were evaluated by one-way ANOVA analysis or unpaired Student *t*-test. Difference with a *p*-value less than 0.05 was considered to be statistically significant.

## 5. Conclusions

In the present study, we introduced the anti-cancer activity of TRPV1 antagonists in human ovarian cancer cells. The novel TRPV1 antagonist, DWP05195, induced caspase-dependent apoptosis. DWP05195 increased the accumulation of intracellular ROS, leading to ER stress through p38 activation. Upon ER stress, the expression of CHOP was upregulated, inducing apoptosis through both intrinsic and extrinsic pathways via the upregulation of BIM and DR4/5.

## Figures and Tables

**Table 1:** Effect of DWP05195 on cell viability in human ovarian cancer cells.

Cell Lines	A2780	SKOV3	OVCAR3	TOV-21G	Hey8A
^a^IC50 (μM)	17.61 ± 1.12	43.87 ± 5.59	78.86 ± 27.05	35.92 ± 7.34	40.83 ± 4.02

^a^IC50 is the concentration that reduces cell number by 50% compared to control cultures. This value represents the average of the results of three independent experiments.

## Supplementary Materials

The following are available online at https://www.mdpi.com/2072-6694/12/6/1702/s1: Table S1: Effect of capsaicin on cell viability in various cancer cells; Table S2: Effect of DWP05195 on cell viability in various cancer cells; Figure S1: Uncropped Western blot figures; Figure S2: Effect of DWP05195 on intracellular calcium levels in human ovarian cancer cell; Figure S3: Involvement of JNK and ERK pathway in DWP05195-induced apoptosis; Figure S4: The expression of TRPV1 in human ovarian cancer cell lines; Figure S5: The expression of TRPV1 in various human cancer cell lines; and Figure S6: Effect of capsaicin on DWP05195-induced apoptosis in human ovarian cancer cells.
