# Supplementary material for: TRPV1 Antagonist DWP05195 Induces ER Stress-Dependent Apoptosis through the ROS-p38-CHOP Pathway in Human Ovarian Cancer Cells
Source: Cancers (Basel). 2020 Jun 26;12(6):1702. doi: 10.3390/cancers12061702 (PMC7352786; doi:10.3390/cancers12061702)
Supplement: Supplementary file 1 [file cancers-12-01702-s001.zip › cancers-827626 Supplementary.pdf]

*Supplementary Materials*

# TRPV1 Antagonist DWP05195 Induces ER Stress-Dependent Apoptosis through the ROS-p38-CHOP Pathway in Human Ovarian Cancer Cells

**Yi-Yue Wang, Kyung-Tae Lee, Myong Cheol Lim and Jung-Hye Choi**

## Materials and Methods

*Cell Lines and Materials*

SH-SY5Y and Caco-2 cell lines were originally obtained from American Type Culture Collection (ATCC; Manassas, VA, USA). Ishikawa cell line was purchased from Sigma-Aldrich (St. Louis, MO, USA). Cells were cultured in DMEM/F12 medium supplemented with 10 % FBS, penicillin (100 U/mL), and streptomycin sulphate (100 μg/mL) in a 5% CO2 - 95% air humidified atmosphere at 37°C. Capsaicin, JNK inhibitor SP600125, and ERK inhibitor PD98059 were purchased from Sigma-Aldrich (St. Louis, MO, USA). Cal 520® AM was obtained from AAT Bioquest, Inc. (Sunnyvale, CA, USA)

*$Ca^{2+}$ Imaging*

A2780 cells were seeded overnight in a 96-well plate. According to manufacturer's instructions, 100 μL of 4μM Cal 520® AM, an intensiometric $Ca^{2+}$ dye, were loaded into the wells, and the cells were incubated at 37°C for 2 h. The dye loading mediums were replaced with DWP05195 (15 μM) or capsaicin (100 μM), then imaged with a confocal time-lapse live cell microscope (K1-Fluo confocal fluorescence laser scanning microscopy; Nanoscope Systems Inc., Daejeon, South Korea) at excitation wavelength of 488 nm and emission wavelength of 510-520 nm. The fluorescence images were captured at intervals of 5 seconds from the video and fluorescence intensity were quantified using Image J.

**Table S1.** Effect of capsaicin on cell viability in various cancer cells.

| Variation | Cell Lines | Capsaicin IC50 (μM) |
|---|---|---|
| **Ovarian Cancer** | A2780 | 120.96 ± 7.96 |
| | SKOV3 | 121.20 ± 19.48 |
| | OVCAR3 | >200 |
| | TOV-21G | >200 |
| | Hey8A | 64.89 ± 6.80 |
| **Neuroblastoma** | SH-SY5Y | 186.36 ± 14.27 |
| **Colon cancer** | Caco-2 | >200 |
| **Endometrial cancer** | Ishikawa | >200 |

**Table S2.** Effect of DWP05195 on cell viability in various cancer cells.

| Cell Lines | SH-SY5Y Neuroblastoma | Caco-2 Colon Cancer | Ishikawa Endometrial Cancer |
|---|---|---|---|
| **DWP05195** IC50 (μM) | 47.32 ± 0.22 | 65.48 ± 6.78 | 82.16 ± 1.31 |

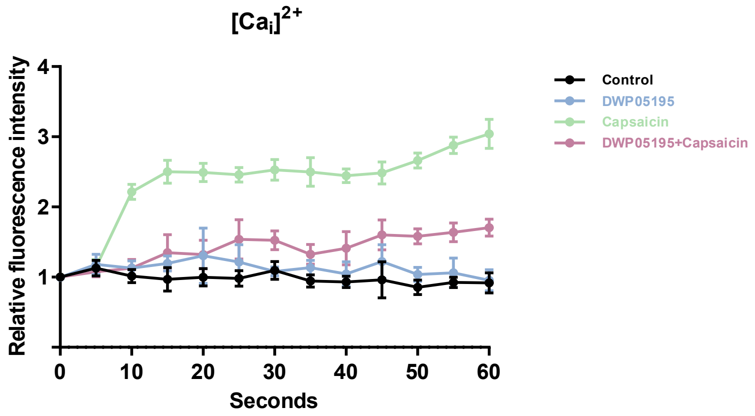


**Figure S2.** Effect of DWP05195 on intracellular calcium levels in human ovarian cancer cells. A2780 cells were seeded overnight in a 96-well plate. After staining with an intensiometric $Ca^{2+}$ dye Cal 520® AM for 2 h, cells were treated with DWP05195 (15 μM) or capsaicin (100 μM). The intracellular calcium levels were observed with time-lapse confocal microscopy. Results are representative of at least three independent experiments.

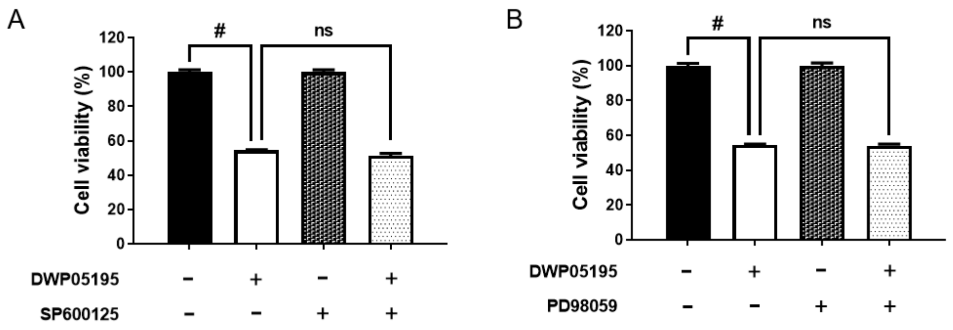


**Figure S3.** Involvement of JNK and ERK pathway in DWP05195-induced apoptosis. A2780 cells were pretreated with (A) JNK inhibitor SP600125 (1 μM) or (B) ERK inhibitor PD98059 (1 μM) for 2 h, and then treated with DWP05195 (15 μM) for 36 h. MTT assay was performed to determine cell viability. Results are representative of at least three independent experiments. *$p < 0.05$.

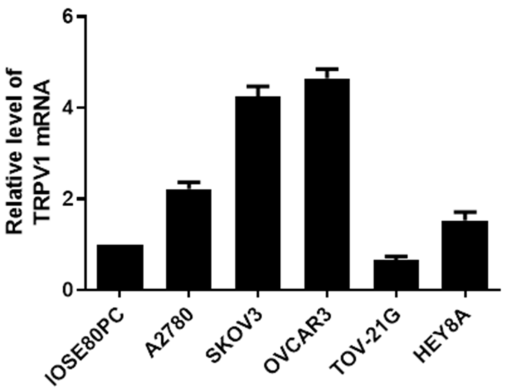


**Figure S4.** The expression of TRPV1 in human ovarian cancer cell lines. The mRNA levels of TRPV1 in immortalized human ovarian surface epithelial cells (IOSE80PC) and human ovarian cancer cell lines (A2780, SKOV3, OVCAR3, TOV-21G, and Hey8A) were analyzed using real-time RT-PCR.

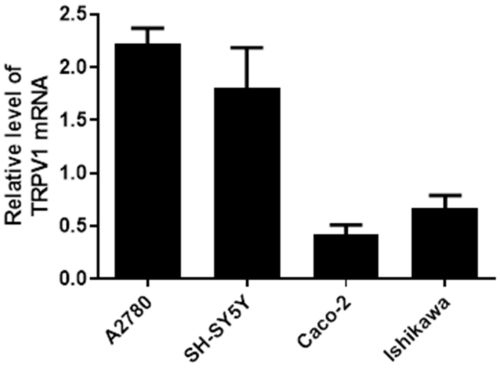


**Figure S5.** The expression of TRPV1 in various human cancer cell lines. The mRNA levels of TRPV1 in human ovarian cancer (A2780), neuroblastoma (SH-SY5Y), colon cancer (Caco-2), and endometrial cancer (Ishikawa) cell lines were analyzed using real-time RT-PCR.

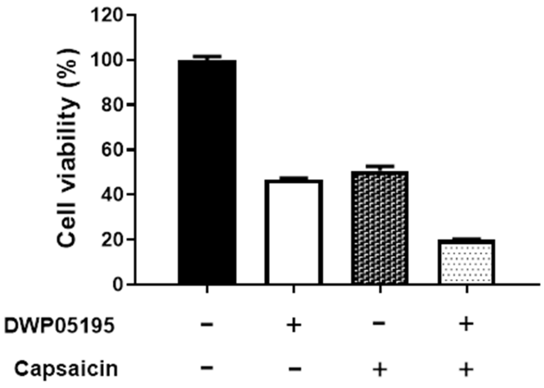


**Figure S6.** Effect of capsaicin on DWP05195-induced apoptosis in human ovarian cancer cells. A2780 cells were pretreated with capsaicin (100 μM), and then treated with DWP05195 (15 μM) for 36 h. MTT assay was performed to determine cell death. Results are representative of at least three independent experiments.

© 2020 by the authors. Licensee MDPI, Basel, Switzerland. This article is an open access article distributed under the terms and conditions of the Creative Commons Attribution (CC BY) license (http://creativecommons.org/licenses/by/4.0/).
