# Supplementary material for: TRPV1 Antagonist DWP05195 Induces ER Stress-Dependent Apoptosis through the ROS-p38-CHOP Pathway in Human Ovarian Cancer Cells
Source: Cancers (Basel). 2020 Jun 26;12(6):1702. doi: 10.3390/cancers12061702 (PMC7352786; doi:10.3390/cancers12061702)

Figure 1F

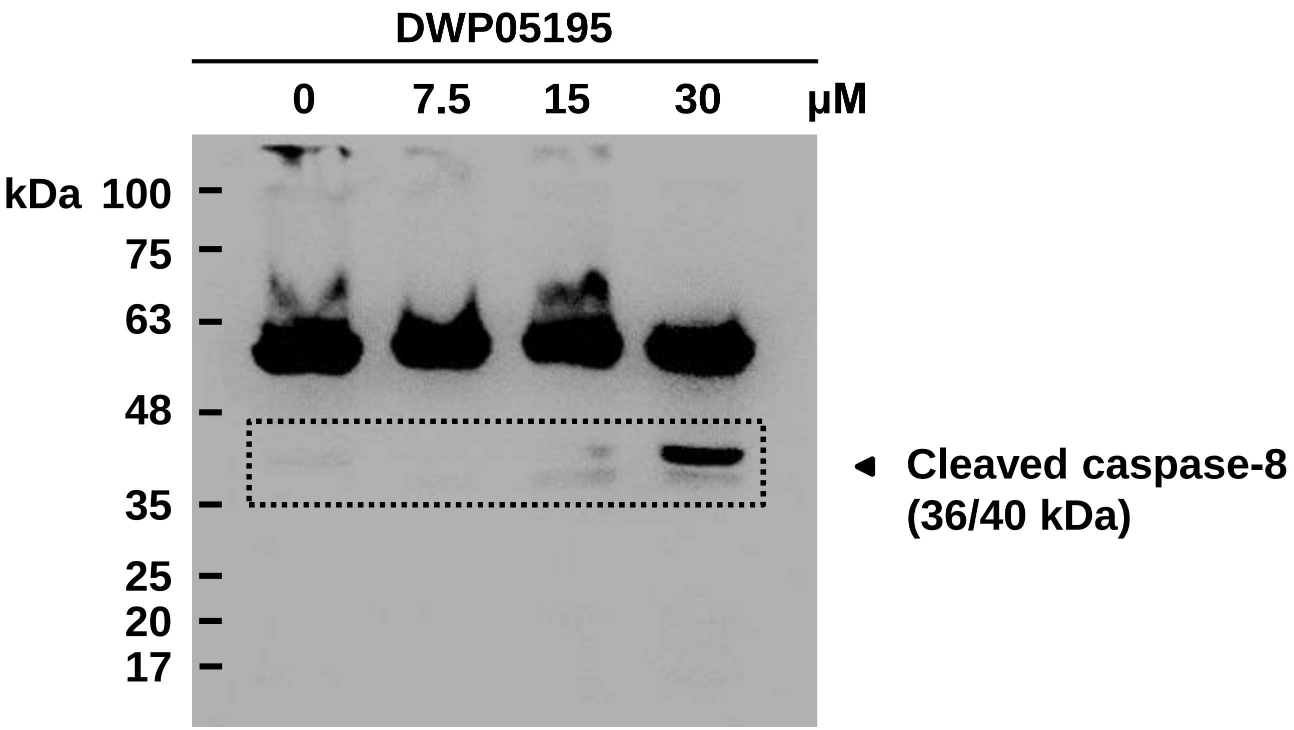

DWP05195
0
7.5
15
30
μM
kDa
100
75
63
48
35
25
20
17
Cleaved caspase-8
(36/40 kDa)

Figure 1F

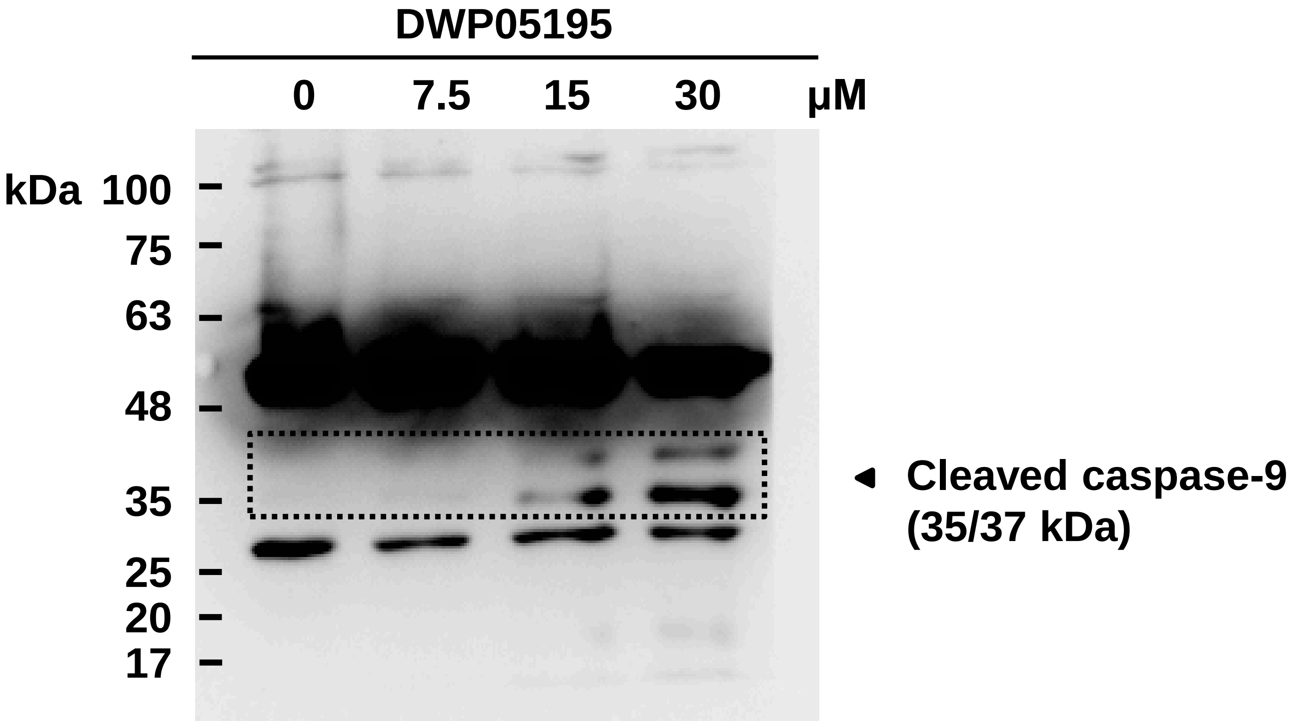
DWP05195
0
7.5
15
30
μM
kDa
100
75
63
48
35
25
20
17
Cleaved caspase-9
(35/37 kDa)

Figure 1F

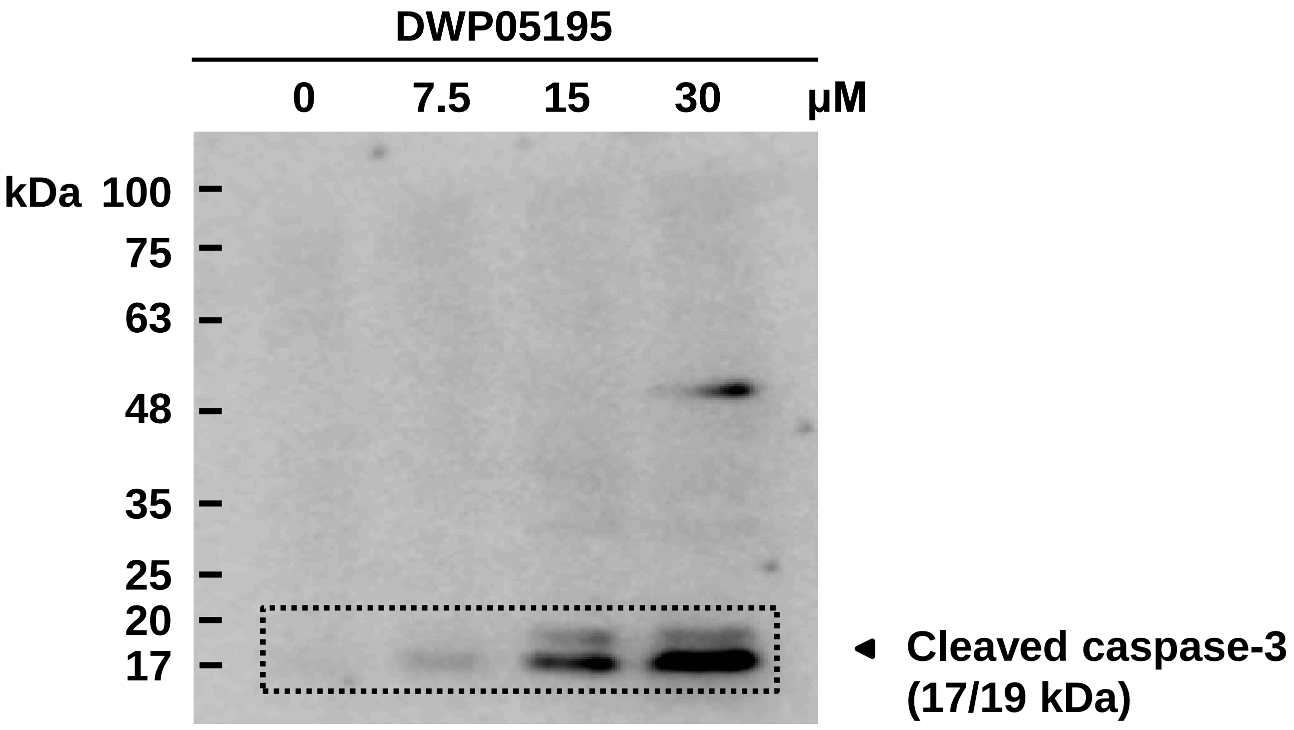
DWP05195
0
7.5
15
30
μM
kDa 100
75
63
48
35
25
20
17
Cleaved caspase-3
(17/19 kDa)

Figure 1F

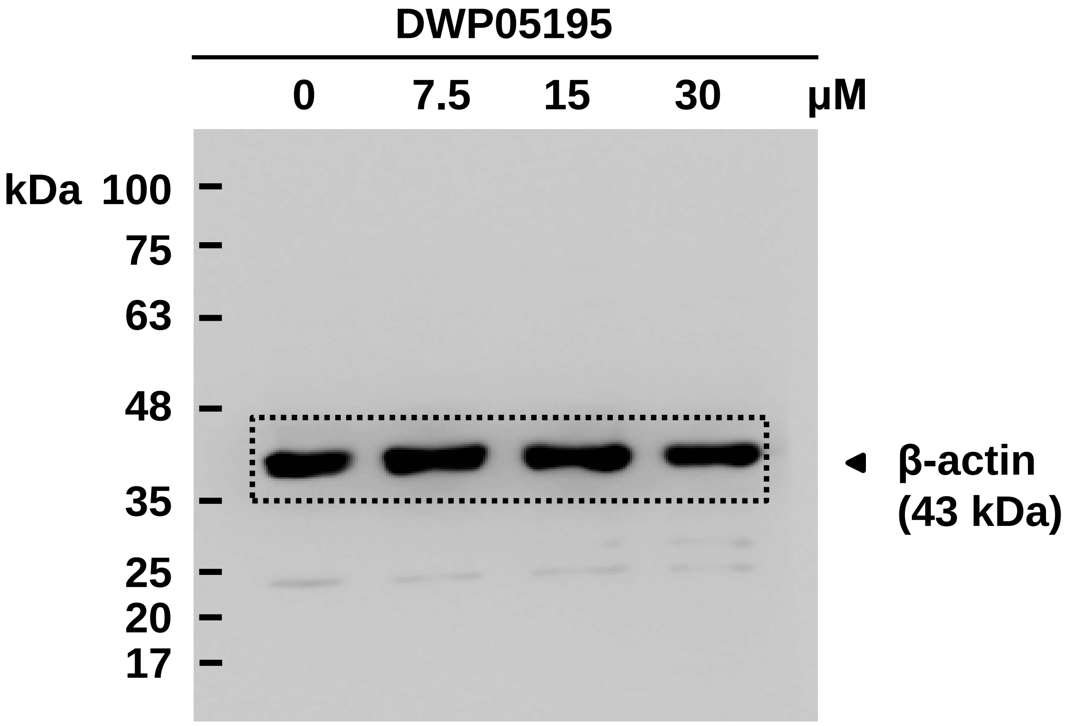

Figure 3A

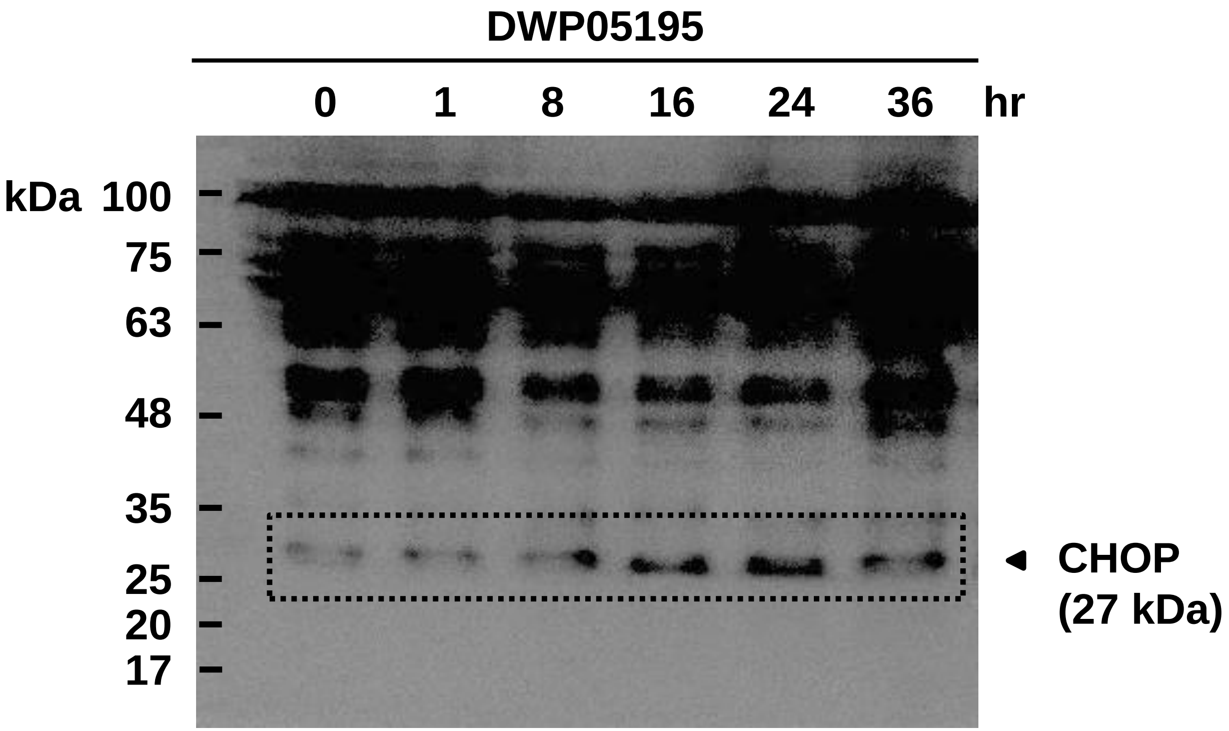

Figure 3A

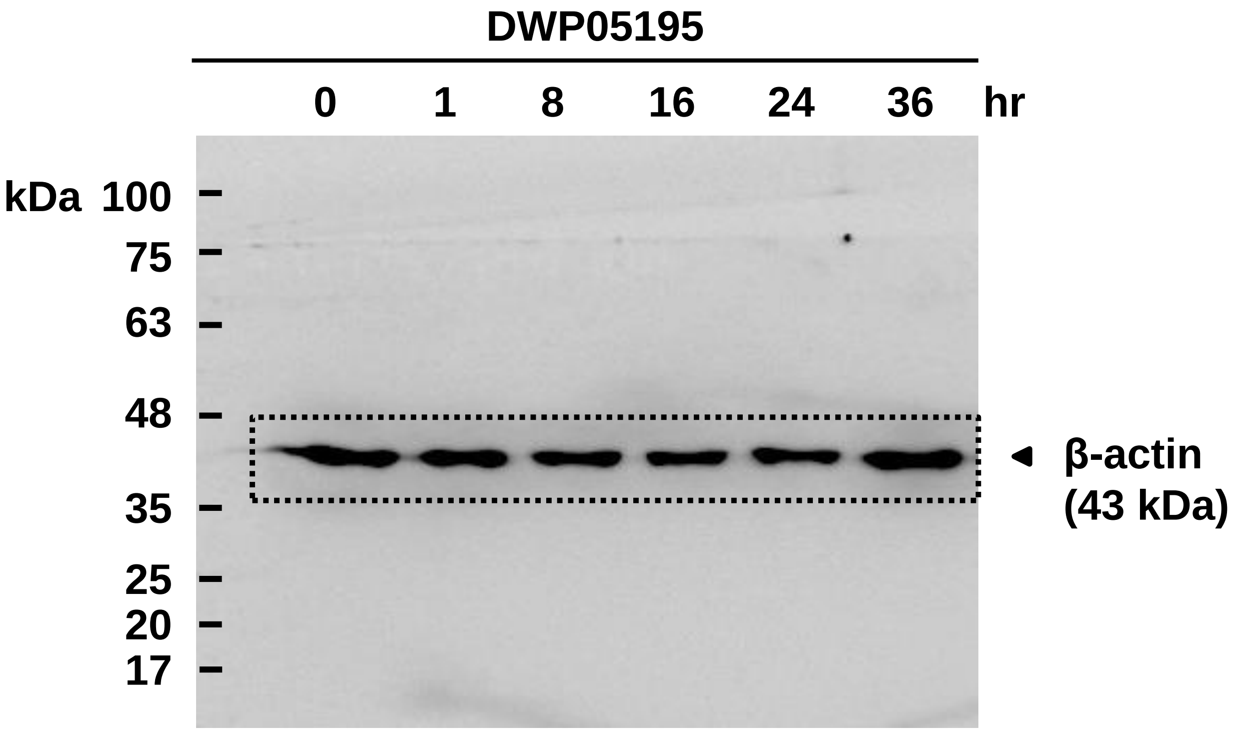
DWP05195
0
1
8
16
24
36
hr
kDa
100
75
63
48
35
25
20
17
β-actin
(43 kDa)

Figure 3C
stable transfection

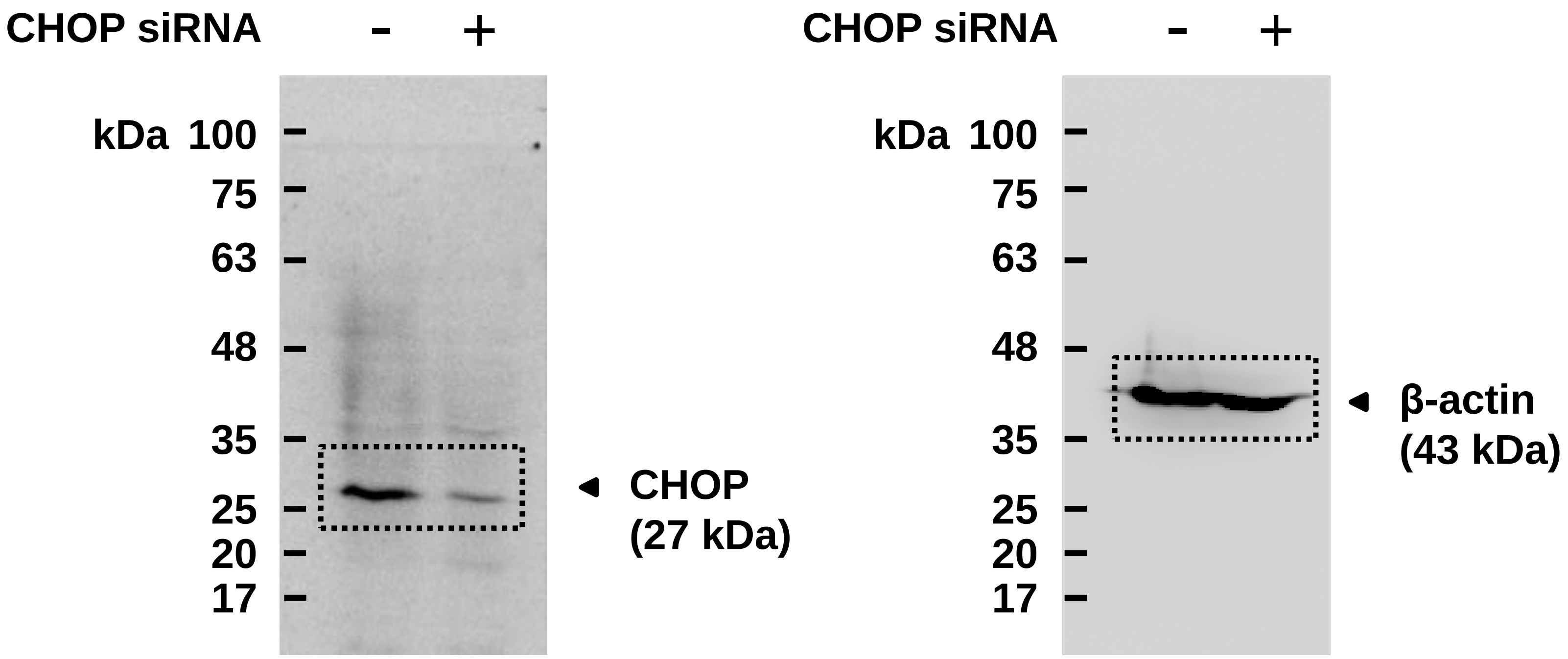

Figure 4C

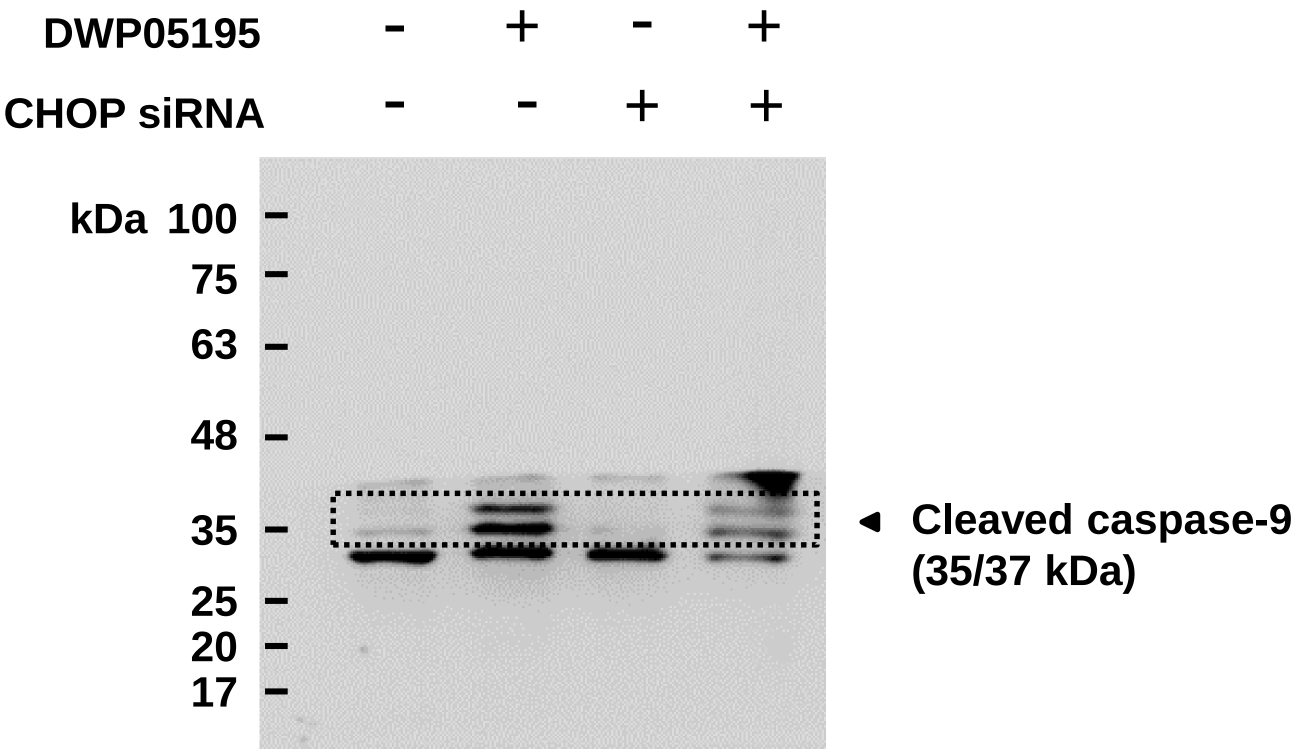
DWP05195 - + - +
CHOP siRNA - - + +
kDa 100
75
63
48
35
25
20
17
Cleaved caspase-9
(35/37 kDa)

Figure 5C

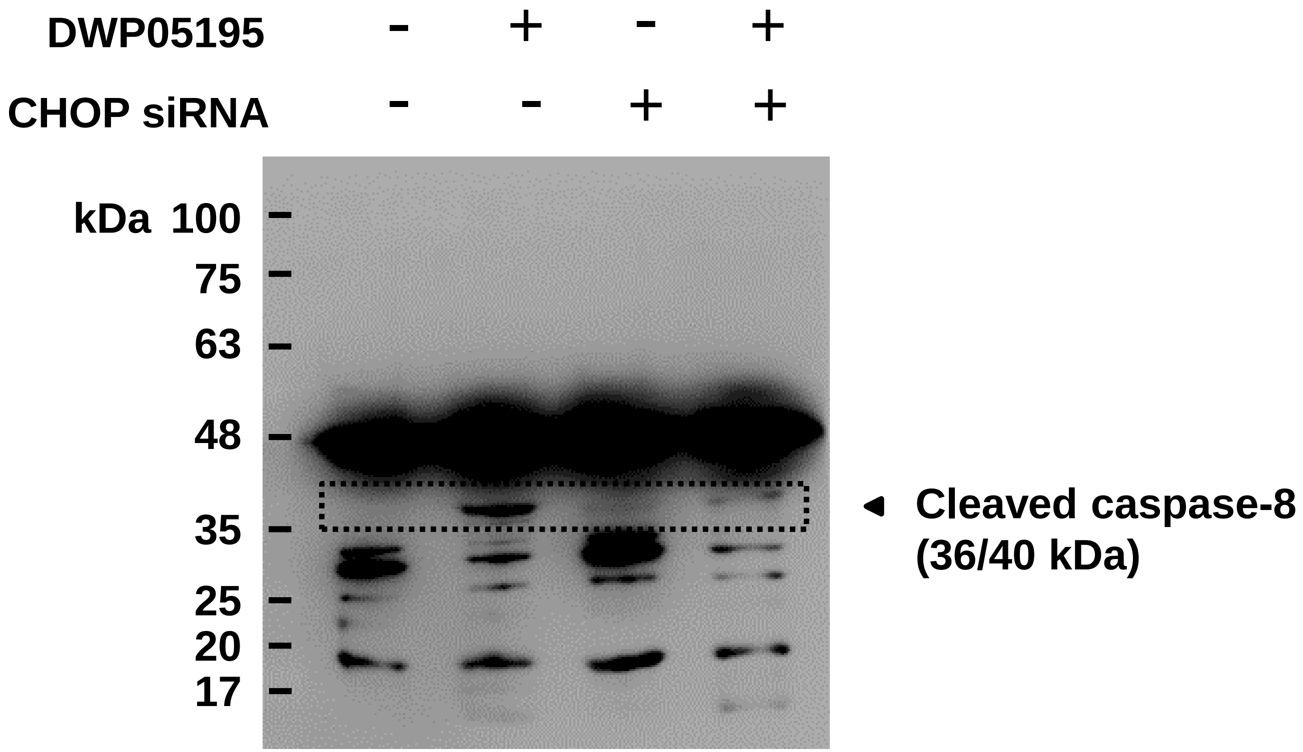

DWP05195 - + - +
CHOP siRNA - - + +
kDa 100
75
63
48
35
25
20
17
Cleaved caspase-8
(36/40 kDa)

Figure 4C, 5C

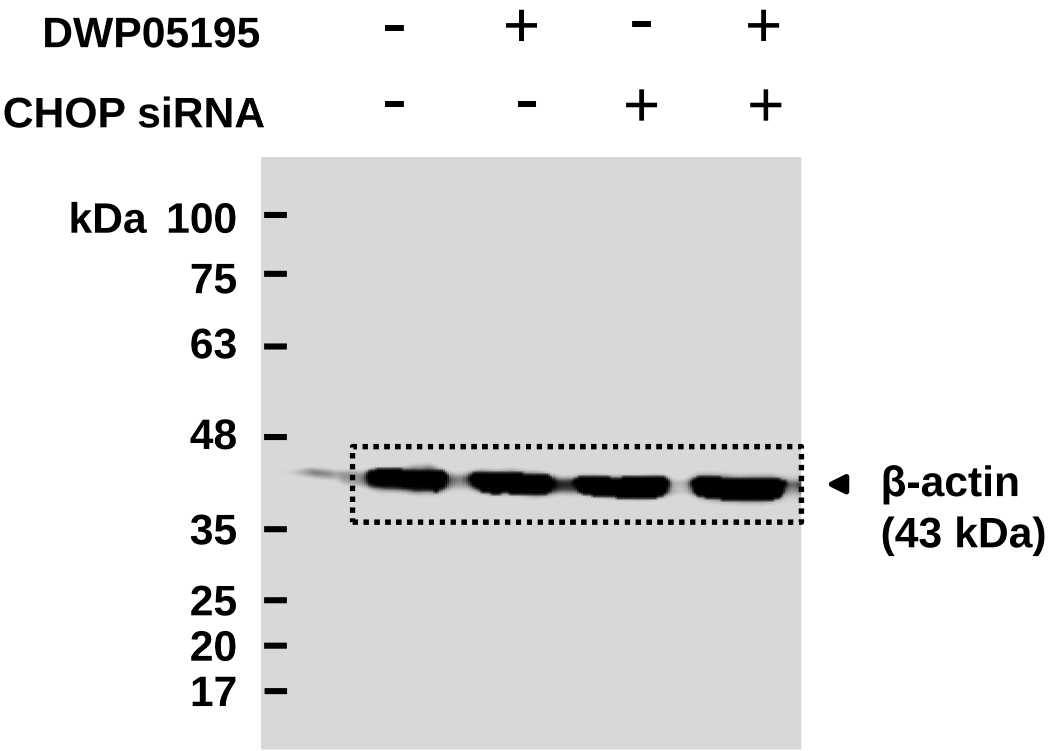

Figure 6C

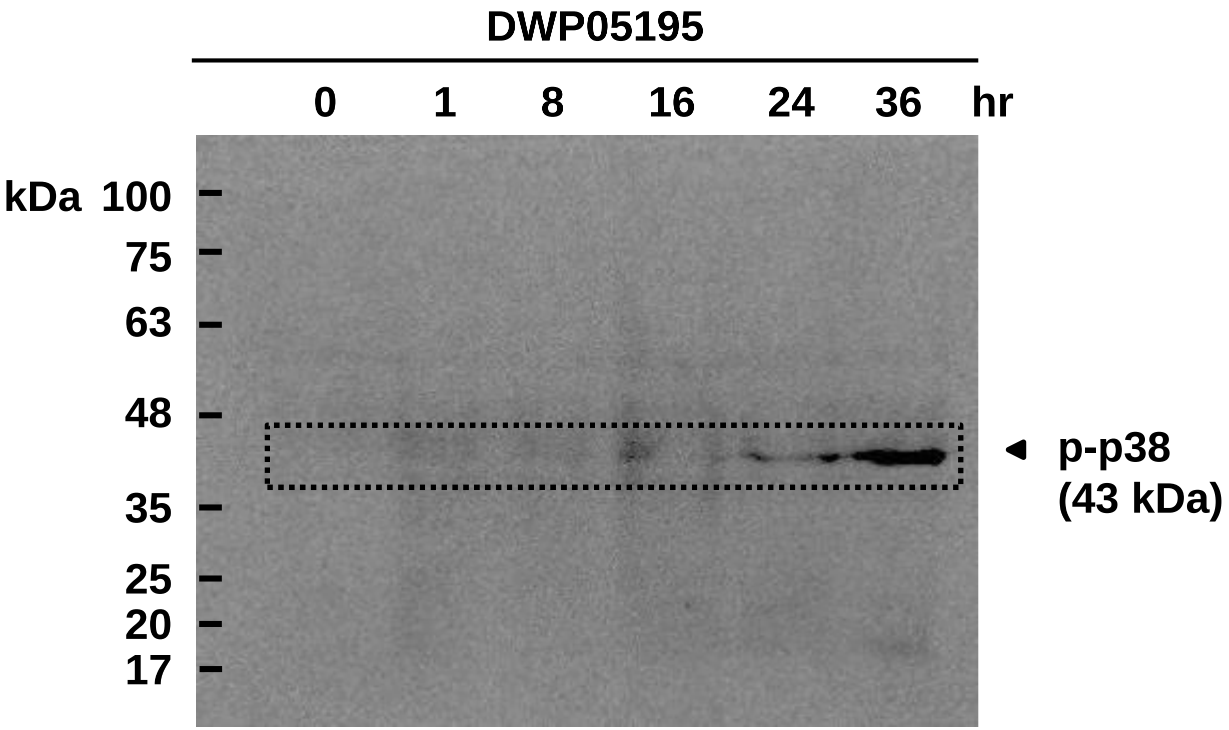

Figure 6C

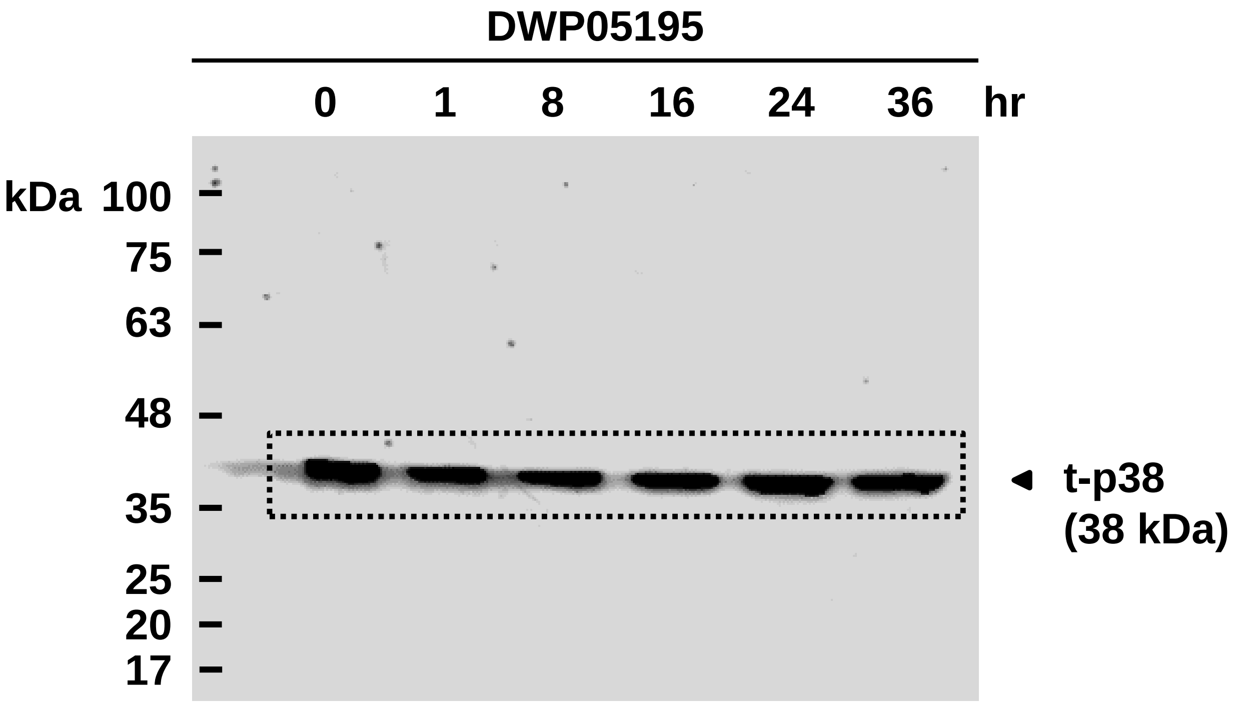

Figure 6C

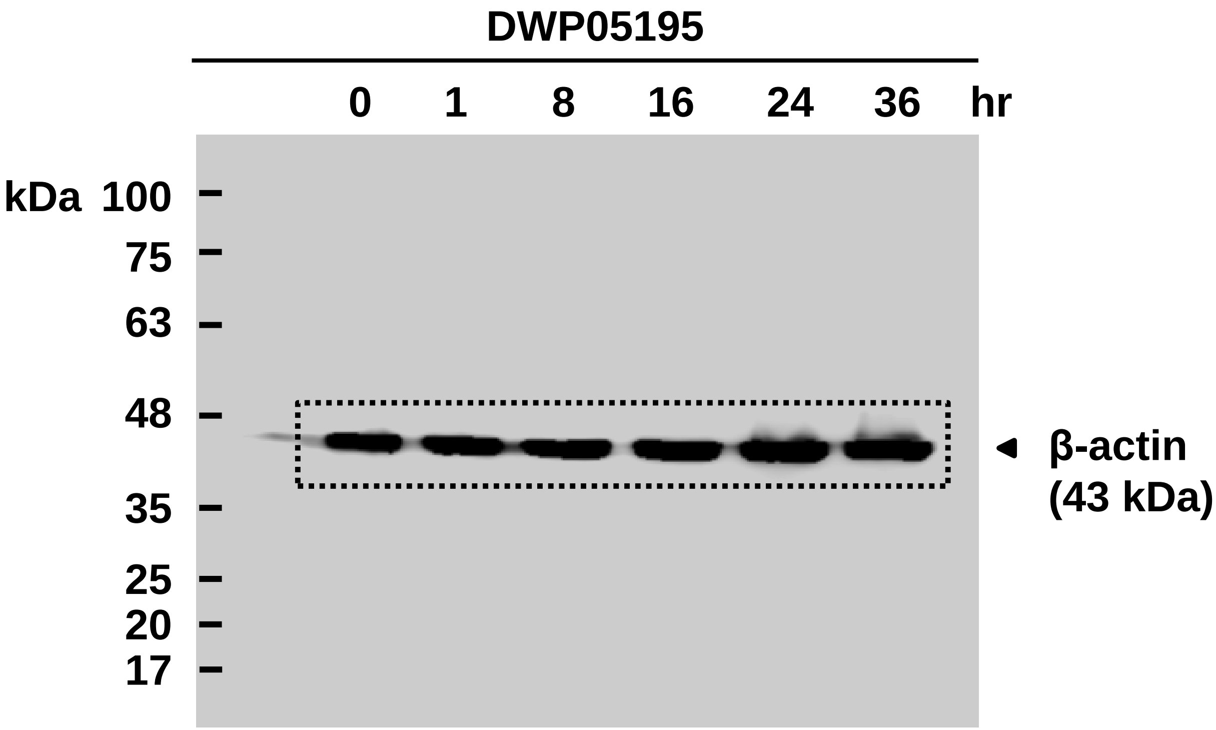

DWP05195
0
1
8
16
24
36
hr
kDa
100
75
63
48
35
25
20
17
β-actin
(43 kDa)

Figure 7A

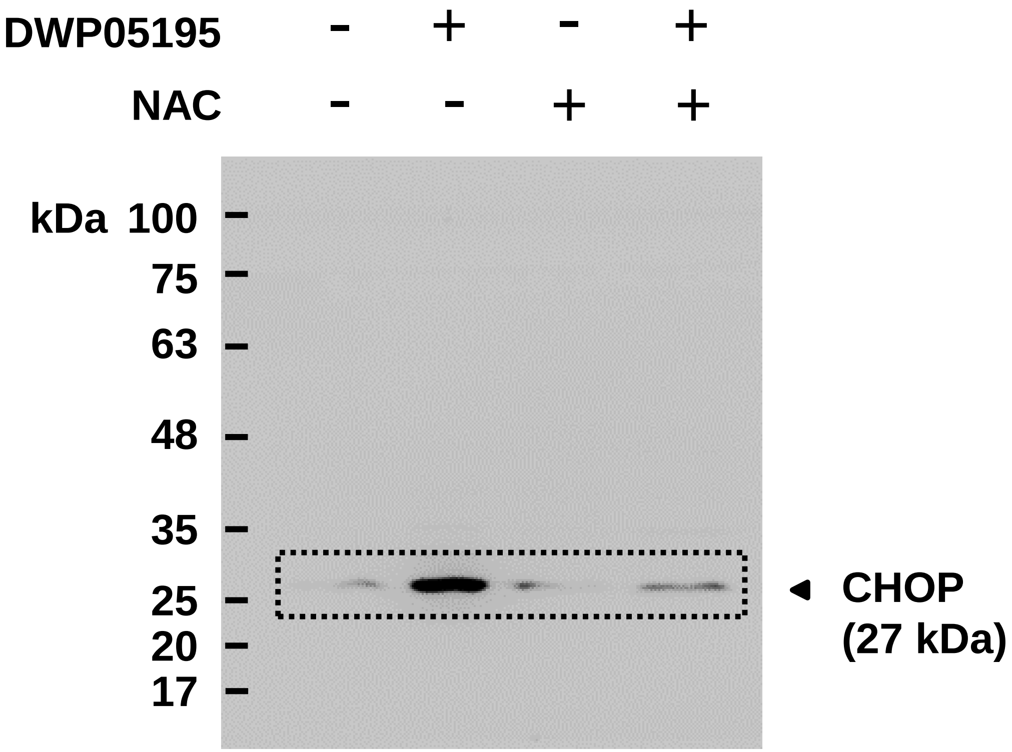

Figure 7A

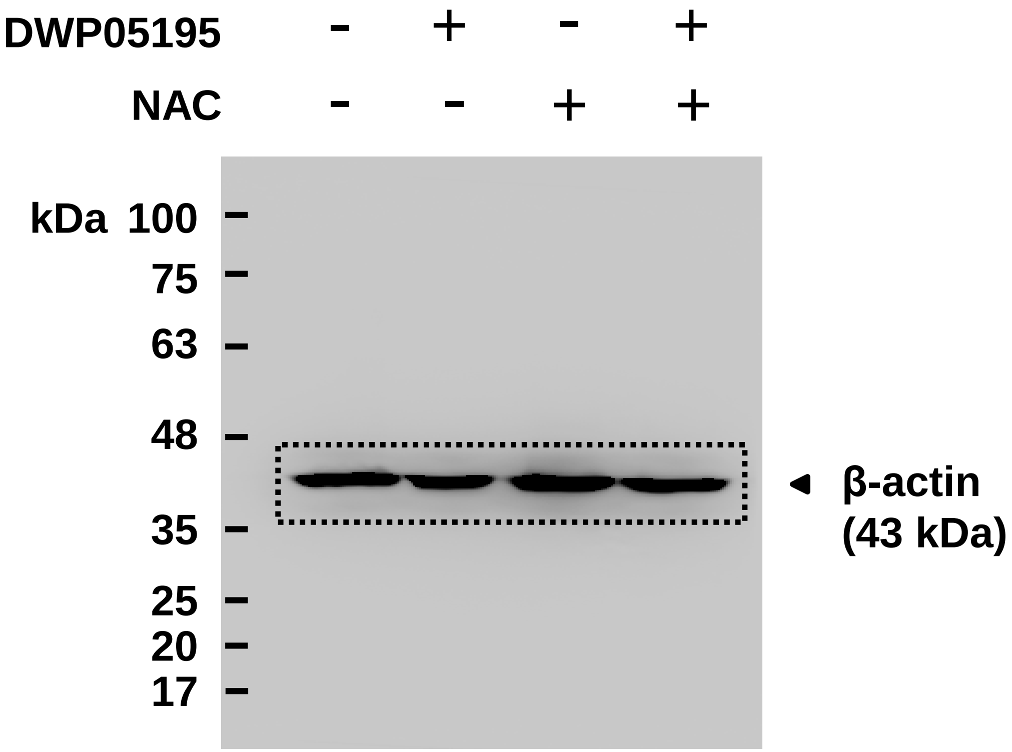

Figure 7B

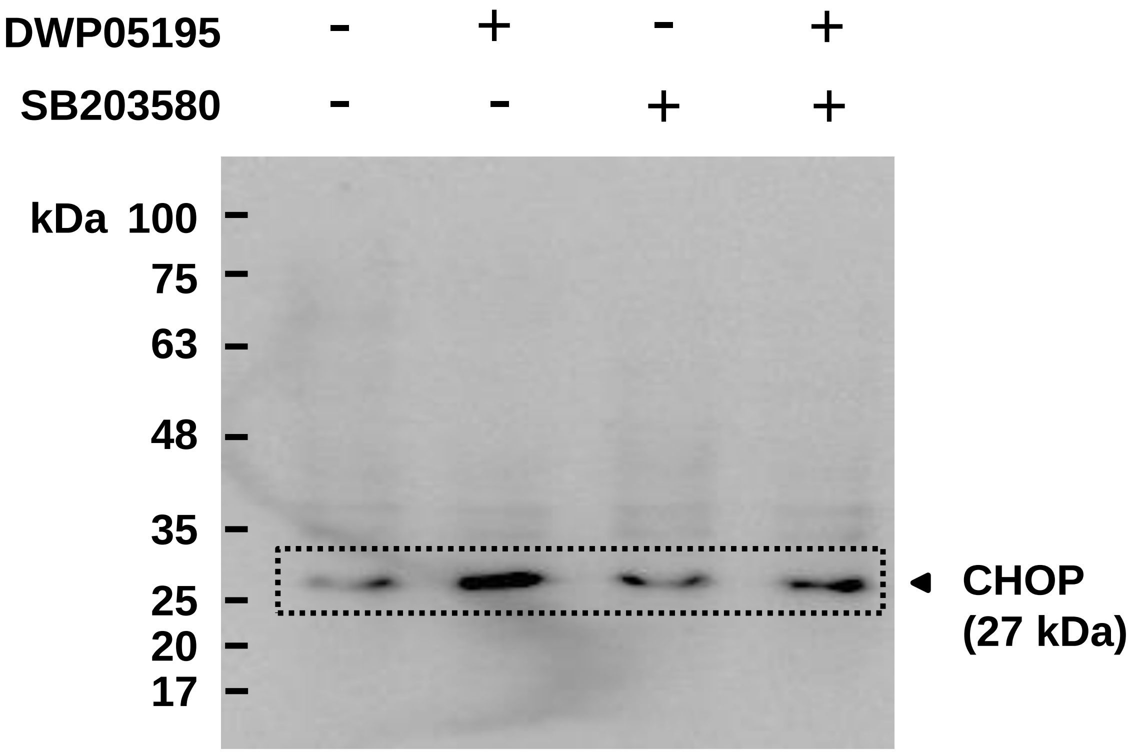

Figure 7B

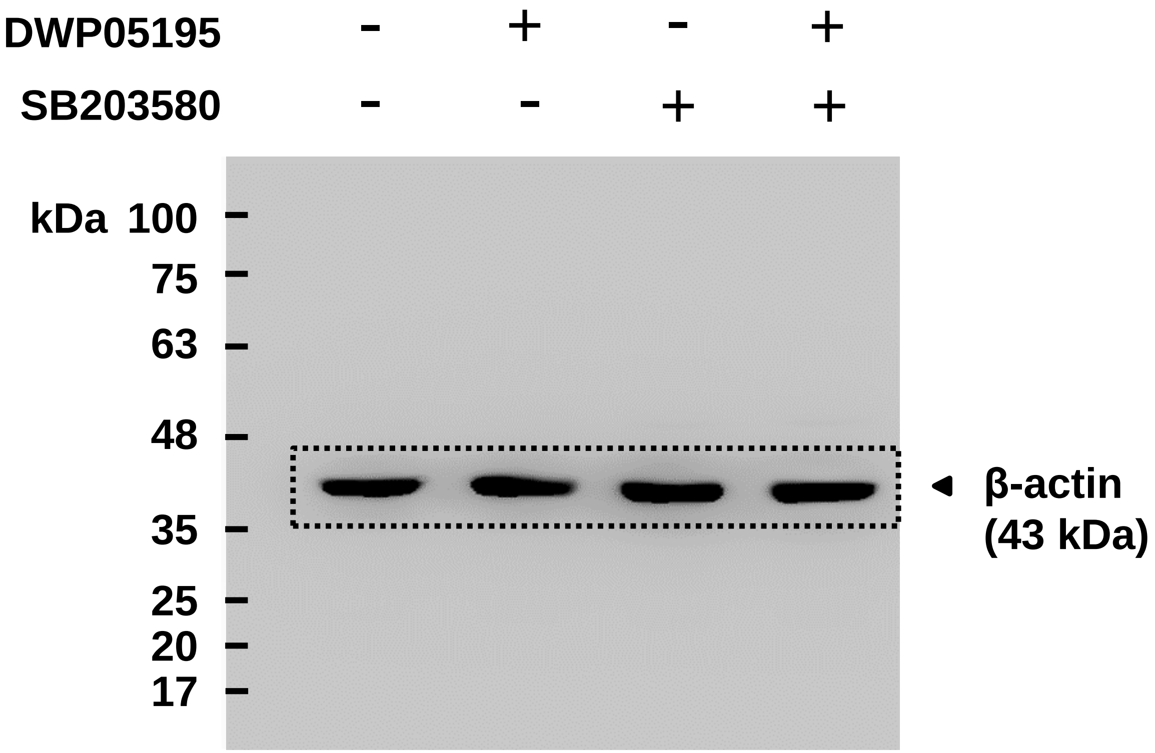

Figure 7C

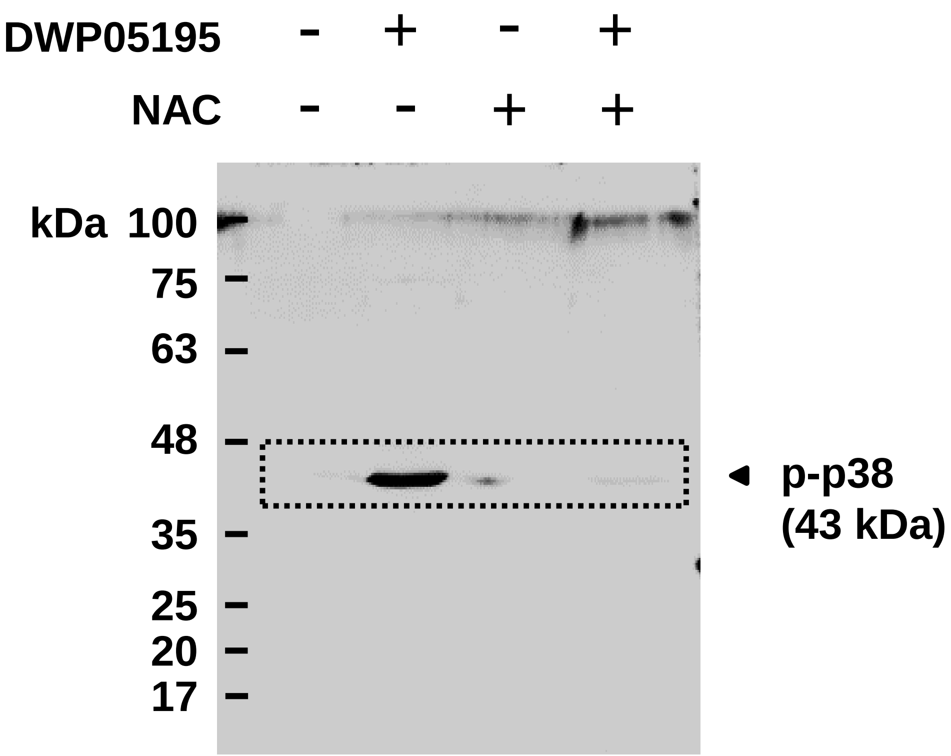

Figure 7C

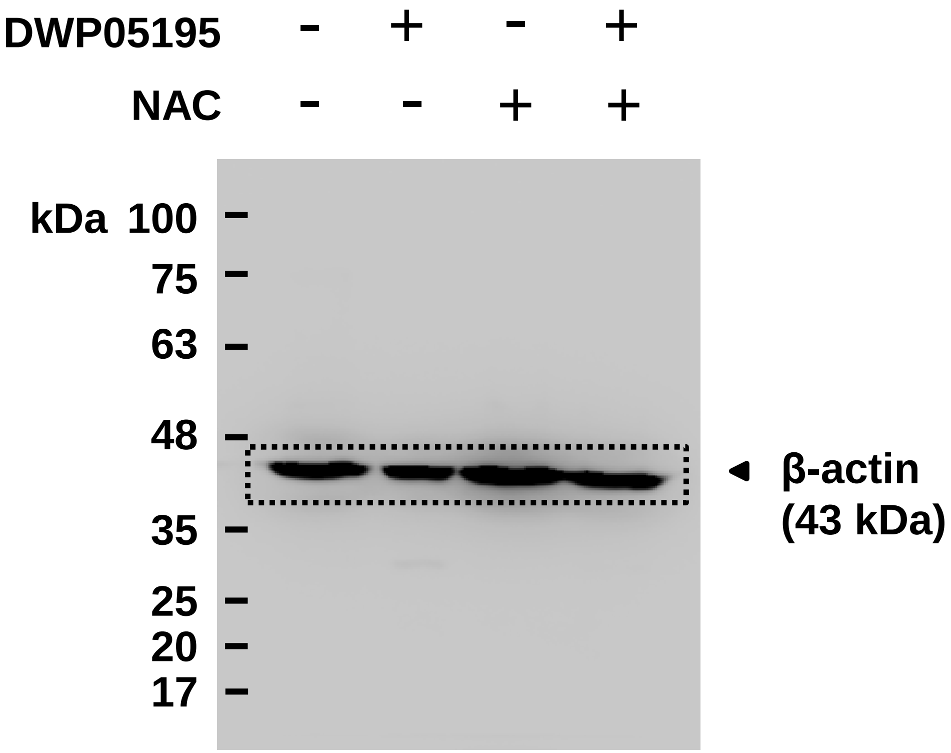

Supplement: Supplementary file 1 [file cancers-12-01702-s001.zip › Figure S1. Uncropped Western blot figures.pdf]
